# ﻿*Clematis danxiacola* (Ranunculaceae), a new species from the Danxia landform area in Zhejiang Province, China

**DOI:** 10.3897/phytokeys.266.154626

**Published:** 2025-11-07

**Authors:** Jun-Ping Li, Qing Ma, Dan-Dan Ma, Jun-Feng Wang, Wen-Yuan Xie, Dong-Hao Wu, Shang-Jiao Ying, Zheng-Hai Chen, Pan Li

**Affiliations:** 1 Zhejiang Yongkang Forestry Bureau, Yongkang 321300, China Zhejiang Yongkang Forestry Bureau Yongkang China; 2 College of Biology and Environmental Engineering, Zhejiang Shuren University, Hangzhou 310015, China Zhejiang Shuren University Hangzhou China; 3 Jiyang College, Zhejiang A&F University, Zhuji 311800, China Zhejiang A&F University Zhuji China; 4 Scientific Research Management Center of East China Medical Botanical Garden, Lishui 323000, China Scientific Research Management Center of East China Medical Botanical Garden Lishui China; 5 Zhejiang Forest Resources Monitoring Centre, Hangzhou 310020, China Zhejiang Forest Resources Monitoring Centre Hangzhou China; 6 Lishui Municipal Administration of Market Supervision of Zhejiang, Lishui 323000, China Lishui Municipal Administration of Market Supervision of Zhejiang Li China; 7 Zhejiang Forestry Survey Planning and Design Co. Ltd., Hangzhou 310020, China Zhejiang Forestry Survey Planning and Design Co. Ltd. Hangzhou China; 8 Key Laboratory of Biodiversity and Environment on the Qinghai Tibetan Plateau, Ministry of Education, School of Ecology and Environment, Xizang University, Lhasa 850000, China Xizang University Lhasa China; 9 Motuo Biodiversity Observation and Research Station of Xizang Autonomous Region, Motuo 860700, China Motuo Biodiversity Observation and Research Station of Xizang Autonomous Region Motuo China

**Keywords:** China, *
Clematis
*, Danxia landform, new species

## Abstract

*Clematis danxiacola*, a novel species of Ranunculaceae discovered in the Danxia landform area of Zhejiang, China, is described and illustrated herein. This species exhibits morphological similarities to *C. terniflora* and *C. chinensis*, but can be distinguished by specific characteristics, including the indumentum of leaflet blades, sepal apex morphology, stamen and pistil quantities, and achene morphology and dimensions. The plastome of *C. danxiacola***sp. nov.** extends 159,506 bp and contains two inverted repeats of 31,040 bp, separated by a large single-copy region of 82,836 bp and a small single-copy region of 18,407 bp. The analysis revealed 136 functional genes, including 92 protein-coding genes, 36 tRNA genes, and 8 rRNA genes. Phylogenetic analyses demonstrated that *C. danxiacola* shares close evolutionary relationship with *C. terniflora* and *C. chinensis*.

## ﻿Introduction

*Clematis* L. (1753: 543) is one of the few cosmopolitan genera in the buttercup family (Ranunculaceae), comprising approximately 300 species predominantly distributed across north temperate zones (Wang 1980; Ziman and Keener 1989; Tamura 1995; Johnson 1997; Grey-Wilson 2000; He et al. 2021). China hosts approximately 147 *Clematis* species, with 93 being endemic (Wang and Bartholomew 2001).

The taxonomy of *Clematis* has been notoriously difficult due to high species-level morphological variation (Johnson 1997; Brandenburg 2000; Grey-Wilson 2000; Wang and Li 2005). Multiple taxonomic schemes have emerged in recent years addressing genus delineation, infrageneric classification, and species delimitation in *Clematis* (Tamura 1995; Johnson 1997; Wang 1998, 2003, Grey-Wilson 2000; Wang and Li 2005; Xiao et al. 2022).

Recent molecular phylogenetic studies suggest substantial convergence in floral and trophic characteristics in *Clematis*, challenging previous infrageneric classifications based on morphological characteristics (Miikeda et al. 2006; Xie et al. 2011), but conforms to Johnson’s broad concept that *Clematis* should encompass all previously recognized genera of subtribe Clematidinae (Johnson, 1997). Currently, ten clades within *Clematis* have been resolved as regional geographic groups (Xie et al. 2011).

During a field survey of the Danxia landform area in Zhejiang, China, we identified an unknown species of *Clematis*. The species displayed morphological similarities to *C. terniflora* DC. (1818: 137), but was notably distinct in habitat, and leaf and floral characteristics, prompting investigation of its taxonomic status.

## ﻿Materials and methods

### ﻿Morphological observation

From 2021 to 2024, we conducted field excursions to the Danxia landform area in Zhejiang Province, China, including Yongkang County, Wuyi County, and Wucheng District (Jinhua); Songyang County, Jinyun County, Qingtian County, and Liandu District (Lishui); Jiangshan County and Kecheng District (Quzhou); Zhuji County and Xinchang County (Shaoxin); and Yuyao County and Fenghau District (Ningbo), to observe and investigate this unknown species. Specimens in the principal herbaria in Zhejiang Province (HHBG, HTC, HZU, and ZM) (Thiers 2024) and specimen photographs from the Chinese Virtual Herbarium (CVH; www.cvh.ac.cn/) were examined to identify specimens similar to the unknown species. High-definition images of similar specimens from international herbaria (A, BM, E, FI, G, K, LD, LINN, MPU, and P) were also consulted. Based on field investigation and herbarium specimen evaluation, the morphology of this new species was documented through comparison with protologues from Greuter (1965) and de Candolle (1818).

### ﻿Sampling and sequencing

#### ﻿Material sampling

From August 10 to August 18, 2023, we collected one leaf sample each from 7 populations of the unknown taxon in Jinhua and Lishui cities of the Danxia landform area in Zhejiang Province (Table 1). Leaves were initially placed in non-woven bags, then stored in sealing bags containing allochroic silica gel for rapid drying. Herbarium specimens were preserved in the herbaria of Zhejiang Museum of Natural History (ZM); Institute of Botany, Chinese Academy of Sciences (PE); and Kunming Institute of Botany, Chinese Academy of Sciences (KUN) (Table 1).

**Table 1.** List of analyzed samples of *Clematis danxiacola* sp. nov.

Molecular specimen ID	Herbarium specimen ID	Locality	Coordinates	Altitude (a.s.l.)
YK-03	ZMNH0067437	Feilongshan, Xicheng Subdistrict, Yongkang County, Jinhua City	120°0'41.40"E, 28°59'23.88"N	181
YK-04	ZMNH0067438	Longqingkeng, Wangxitian Village, Xiangzhu Town, Yongkang County, Jinhua City	120°2'33.06"E, 29°0'8.40"N	246
YK-07	ZMNH0067439	Qianlang Village, Shizhu Town, Yongkang County, Jinhua City	120°7'14.50"E, 28°49'59"N	175
YK-08	ZMNH0067436 PE02621603 KUN1644125	Chiyantang Reservoir, Xianling Village, Zhiying Town, Yongkang County, Jinhua City	120°8'7.86"E, 28°54'29.28"N	172
LD-01	ZMNH0067440	Nanbenjis, Baiyunshan, Baiyun Subdistrict, Liandu District, Lishui City	119°55'4.71"E, 28°28'54.4"N	176
LD-02	ZMNH0067441	Huangnidan Village, Zijin Subdistrict, Liandu District, Lishui City	119°57'47.73"E, 28°27'37.23"N	98
LD-03	ZMNH0067442	Huangcun Village, Huangcun Town, Liandu District, Lishui City	120°2'48.74"E, 28°30'40.02"N	129

#### ﻿DNA extraction, PCR amplification, and sequencing

Whole-genomic DNA from silica-dried leaf tissue was extracted using a modified CTAB protocol (Chen et al. 2014). For plastome sequencing, high-quality DNA was fragmented into lengths of ≤800 bp. Fragment quality was verified using an Agilent Bioanalyzer 2100 (Agilent Technologies, Palo Alto, CA, USA). A short-insert (500 bp) paired-end library was constructed and sequenced by Nanopore Technology (Wuhan, China) on a DNBSEQ-T7 sequencer with 150 bp paired-end reads.

For amplification and sequencing of ribosomal DNA (rDNA) internal transcribed spacer (ITS) regions, primers were designed using ITS sequences of closely related species: forward, 5′-ATGCGATACTTGGTGTGAAT-3′; reverse, 5′-GACGCTTCTCCAGACTACA-3′. The amplification fragments encompass the ITS1, ITS2, 5.8 rDNA, and 26S rDNA regions. DNA amplification was conducted in 50 μL reactions containing 1 μL of total DNA (20 ng/μL), 5 μL of 10× buffer with 2.5 mM Mg^2+^, 1 μL of dNTP (10 mM), 1.5 μL of each primer (10 μM), and 1 μL of Taq polymerase (5 u/μL) in an ABI-2720 thermocycler (Applied Biosystems, Waltham, MA, USA). The PCR protocol consisted of initial denaturation at 95 °C for 5 min, followed by 35 cycles of denaturation at 95 °C for 30 s, annealing at 58 °C for 30 s, and extension at 72 °C for 90 s, with a final extension at 72 °C for 7 min. PCR products were purified by ethanol precipitation. The purified products were analyzed by electrophoresis on a 1% (w/v) agarose gel and spectrophotometric analysis (NanoDrop 2000; Thermo Fisher Scientific, Waltham, MA, USA) before Sanger sequencing using an ABI3730-XL sequencer (Applied Biosystems).

### ﻿Data assembly and annotation

For plastome assembly and annotation, raw sequencing data was filtered using FastQC (www.bioinformatics.babraham.ac.uk/projects/fastqc/). Clean paired-end reads were *de novo* assembled using the GetOrganelle pipeline (Jin et al. 2020) using SPAdes 3.10.1 as the assembler (Bankevich et al. 2012). The plastome of *C. terniflora* (NC028000) served as the reference. The assembled plastome was imported into Geneious Prime (https://www.geneious.com) for annotation and identification of putative starts, stops, and intron positions through comparison with homologous genes of published *Clematis* plastomes. The circular plastome map of the new species was generated using OGDRAW (http://ogdraw.mpimp-golm.mpg.de/) (Greiner et al. 2019).

### ﻿Phylogenetic analyses

For phylogenetic analyses, complete plastome sequences of 85 *Clematis* species and ITS sequences of 80 *Clematis* species were obtained from GenBank (Suppl. material 1: table S1). The sampling strategy encompassed all major clades within *Clematis* previously identified (Xie et al. 2011; Xiao et al. 2022). The plastome and ITS dataset were aligned independently using multiple alignment using fast Fourier transform (MAFFT) v7.490 implemented in Geneious Prime (Katoh and Standley 2013). Phylogenetic analyses based on ITS and plastome sequences (excluding one copy of the inverted repeat) were conducted to explore the evolutionary relationship among the new species and other *Clematis* species using the maximum likelihood method with RAxML-HPC v8.2.12 (Stamatakis 2014) on the CIPRES Science Gateway (http://www.phylo.org/). According to Jiang et al. (2017) and Xiao et al. (2022), *Anemoclema glaucifolium* (Franch.) W.T. Wang (Ranunculaceae) was chosen as an outgroup.

### ﻿Morphological Analysis

Morphological characteristics were documented from 10 wild individuals of *C. danxiacola* sp. nov. during field surveys. For comparative analysis, 10 herbarium specimens each of *C. terniflora*, *C. chinensis*, and C. chinensis var. vestita were examined from the Chinese Virtual Herbarium (CVH; https://www.cvh.ac.cn/).

Twenty quantitative traits (Table 2) were selected to construct a morphological matrix. Principal component analysis (PCA) was performed for dimensionality reduction, and a multidimensional morphospace was established based on the first two principal components. All computations were conducted using GraphPad Prism v10.1.2 (Build 324).

**Table 2.** PCA factor loadings based on 20 morphological characteristics.

Variable	Data Type	PC1	PC2	PC3
Specimen color after drying (blackened 1 / unblackened 0)	DV	0.808	−0.366	0.121
Compound leaf length (cm)	CV	−0.46	0.185	0.564
Compound leaf width (cm)	CV	−0.605	0.162	0.539
Compound leaf length/width ratio	CV	0.42	−0.093	−0.105
Indumentum on leaf abaxial surface (densely pubescent 1 / glabrous or glabrescent 0)	DV	−0.643	−0.612	−0.187
Terminal leaflet length (cm)	CV	−0.693	−0.249	0.47
Terminal leaflet width (cm)	CV	−0.215	−0.064	0.367
Terminal leaflet length/width ratio	CV	−0.548	−0.256	0.303
Petiolule length (cm)	CV	−0.024	0.563	0.361
Pedicel length (cm)	CV	0.158	0.128	0.256
Calyx apex morphology (truncate 1 / mucronate 0)	DV	−0.879	−0.399	−0.048
Calyx length (mm)	CV	−0.4	0.596	0.254
Calyx width (mm)	CV	−0.764	0.194	−0.3
Calyx length/width ratio	CV	0.403	0.346	0.56
Stamen number	CV	0.379	0.262	−0.048
Fruit length (mm)	CV	−0.289	0.609	−0.312
Persistent style length (cm)	CV	0.502	−0.279	0.386
Fruit length/persistent style ratio	CV	−0.296	0.512	−0.508
Taste of fresh leaves (pungent 1 / non-pungent 0)	DV	−0.808	0.366	−0.121
Habitat (ridge, hillside 1 / valley, plain or wetland 0)	DV	−0.879	−0.399	−0.048

DV: dummy variable; CV: continuous variable

## ﻿Results

### ﻿Characteristics of the plastome

**Figure 1.**
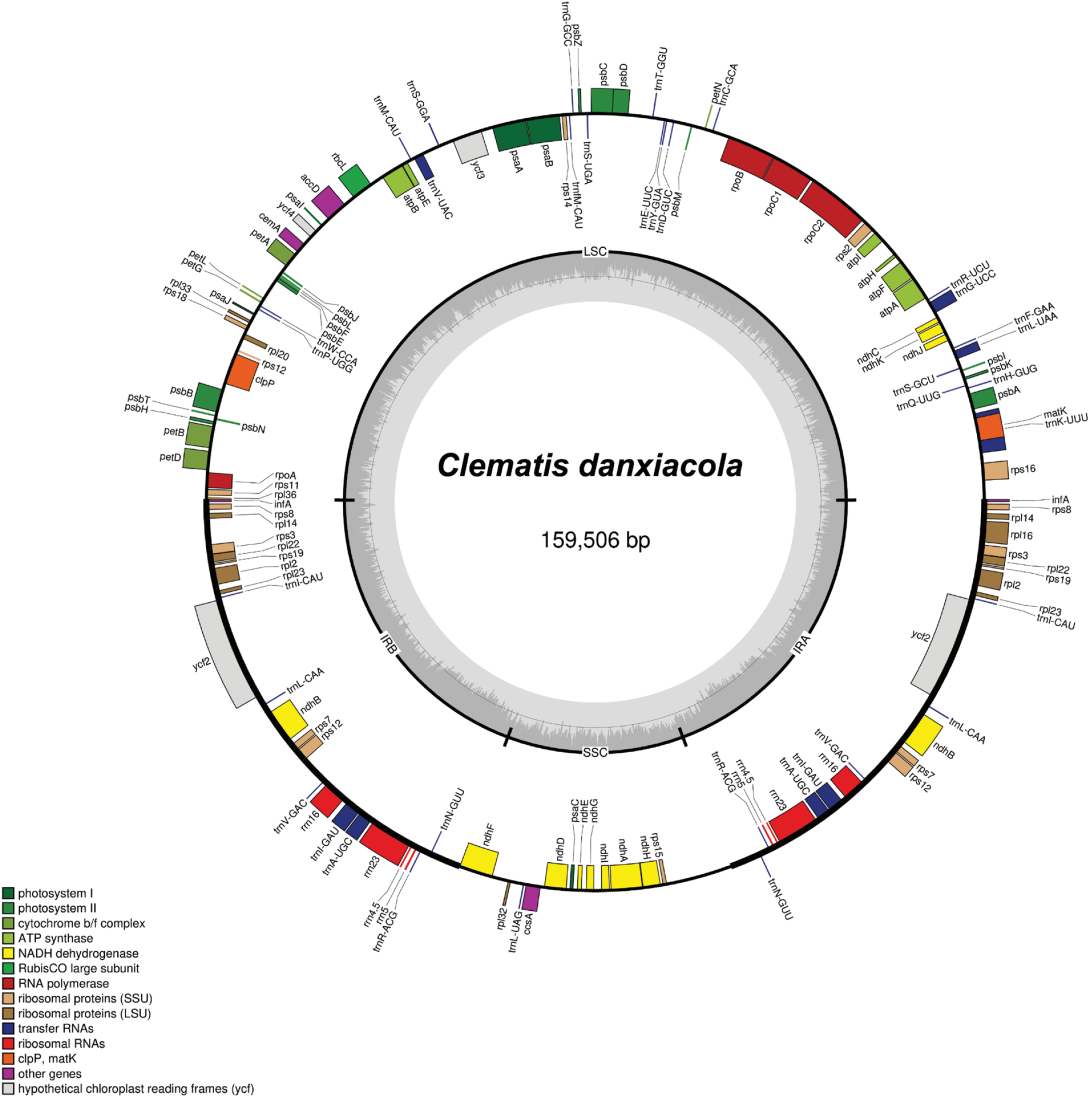
Plastome map of *Clematis danxiacola* sp. nov. Genes inside the circle are transcribed clockwise, while those outside are transcribed counterclockwise. In the inner circle, light gray represents the AT content, and dark gray represents the GC content. Different colors indicate genes belonging to distinct functional groups.

The plastome size of *Clematis danxiacola* sp. nov. is 159,506 bp and comprises two 31,040 bp inverted repeats, a 18,407 bp small single-copy region, and a 82,836 bp large single-copy region. The overall GC content is 38%, and the GC contents of the large single-copy, small single-copy, and inverted repeat regions are 36.3%, 31.4%, and 42.0%, respectively. The complete plastome contains 136 functional genes, including 92 protein-coding genes, 36 tRNA genes, and 8 rRNA genes (Fig. 1, Suppl. material 1: table S2). The complete plastome sequence of *C. danxiacola* after annotation was deposited in GenBank (PQ246280).

### ﻿Molecular analysis

**Figure 2.**
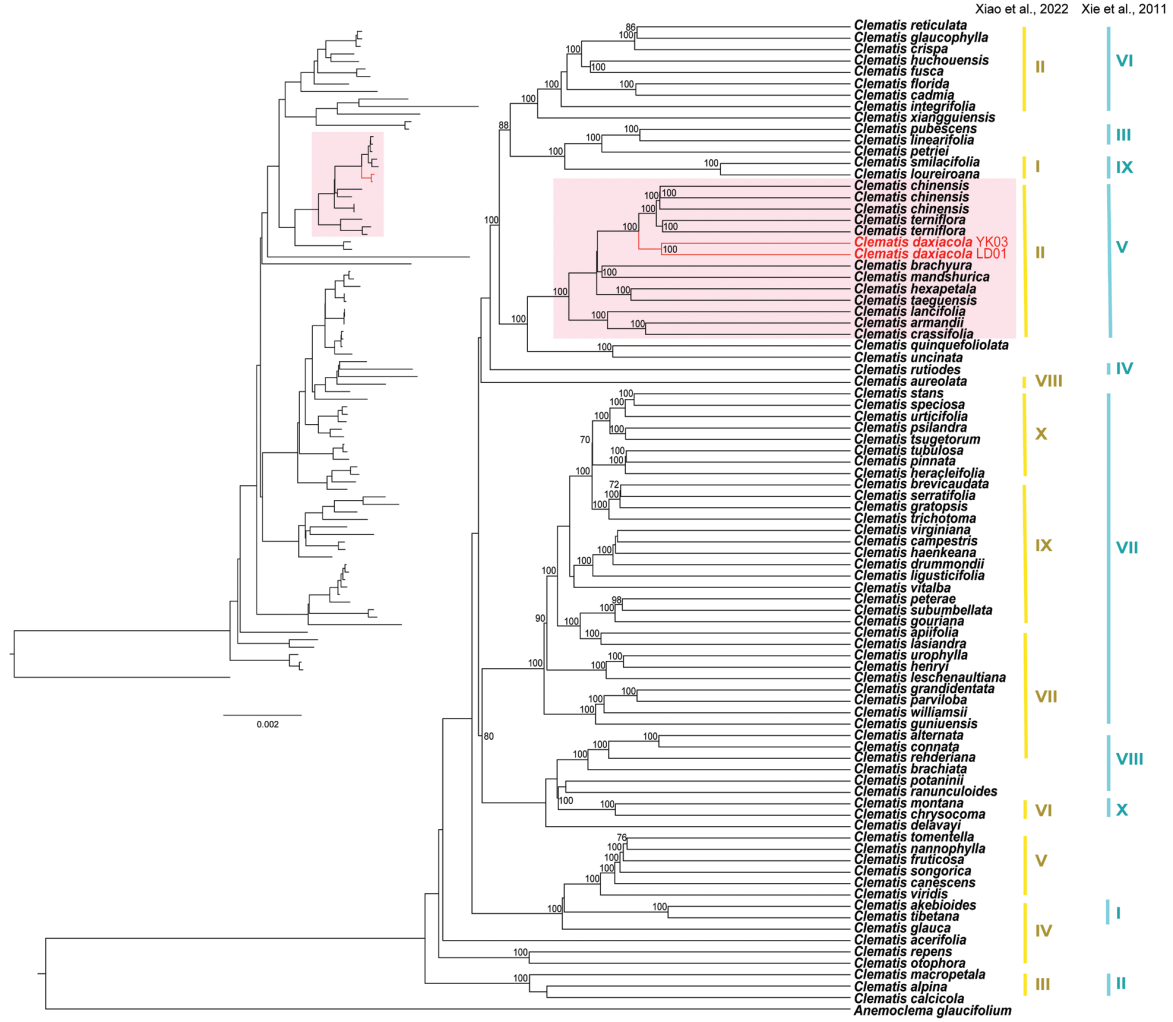
Maximum likelihood tree inferred from *Clematis* plastomes to elucidate the phylogenetic position of *C. danxiacola* sp. nov. Numbers above the branches indicate the bootstrap values (≥50%) of the maximum likelihood analysis. Branches and names in red indicate the new species (*C. danxiacola* sp. nov). Light red shading highlights the clade containing the new species, as revealed by phylogenetic analyses in Xie et al. (2011) and Xiao et al. (2022). Numerals on the right indicate the ten *Clematis* clades recognized in Xie et al. (2011) and Xiao et al. (2022): yellow, Xiao et al. (2022); blue, Xie et al. (2011).

**Figure 3.**
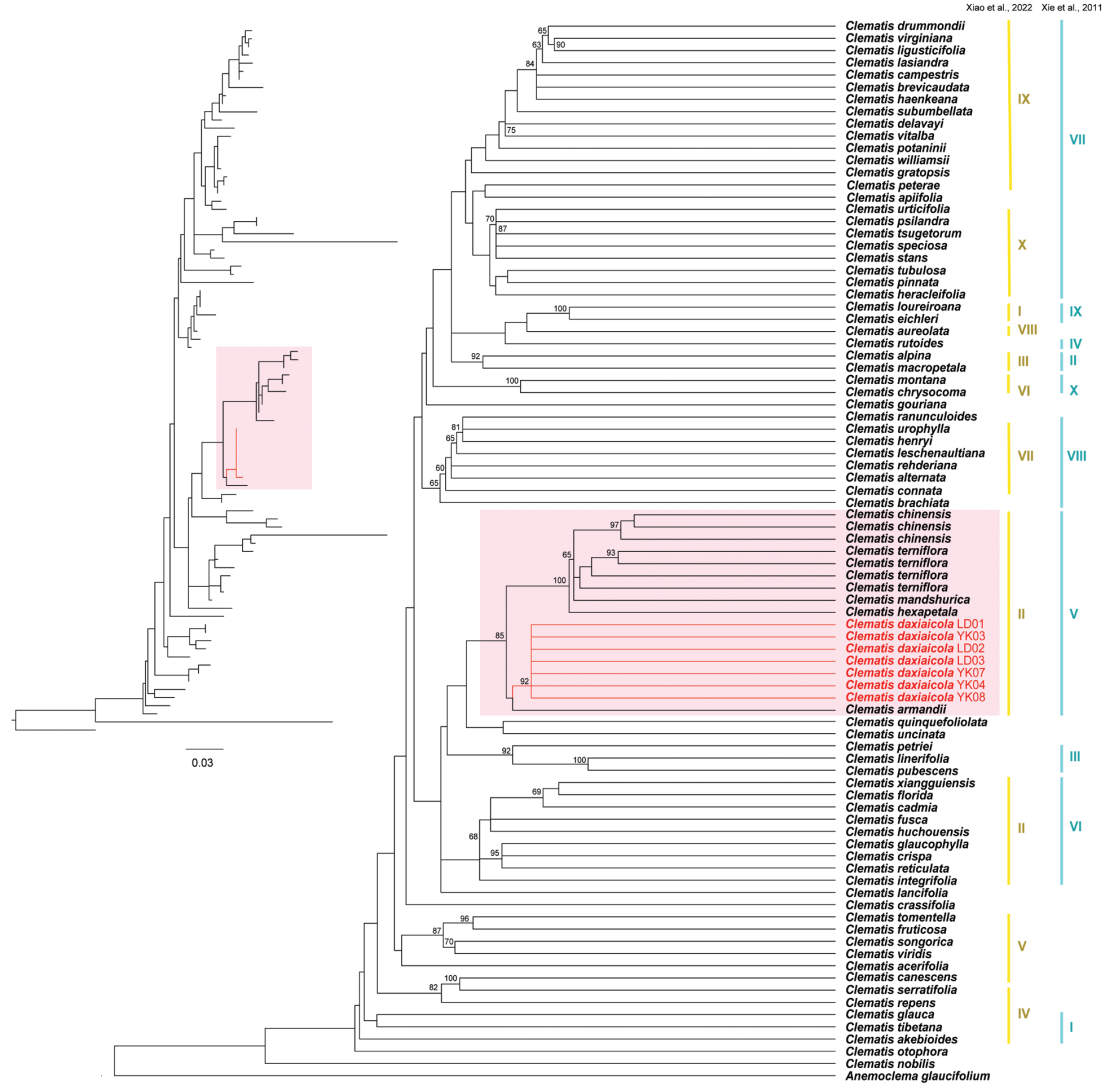
Maximum likelihood tree inferred from ITS sequences to elucidate the phylogenetic position of *C. danxiacola* sp. nov. Numbers above the branches indicate the bootstrap values (≥50%) of the maximum likelihood analysis. Branches and names in red indicate the new species (*C. danxiacola* sp. nov). Light red shading highlights the clade containing the new species, as revealed by phylogenetic analyses in Xie et al. (2011) and Xiao et al. (2022). The GenBank accession numbers used for the analysis are indicated after the species names. Numerals on the right indicate the ten *Clematis* clades recognized in Xie et al. (2011) and Xiao et al. (2022): yellow, Xiao et al. (2022); blue, Xie et al. (2011).

Phylogenetic analyses based on both plastome and ITS datasets revealed that individuals of *C. danxiacola* were clustered with high bootstrap support values (Figs 2–3). The new species is closely related to *C. terniflora* and *C. chinensis*Osbeck (1757: 205) and belongs to clade V (sensu Xie et al. 2011) or clade II (sensu Xiao et al. 2022) of *Clematis*, which comprises species of sect. Flammula DC., sect. Viticella DC., and sect. Viorna (sensu Tamura 1995).

### ﻿Morphological comparison

Morphological comparisons between *C. danxiacola* sp. nov., *C. terniflora*, *C. chinensis*, and C. chinensis var. vestita (Rehder & E.H.Wilson) W.T. Wang (1998: 158) are presented in Table 2. This new species shares characteristics with *C. terniflora* and *C. chinensis* in having climbing stems, entire leaflet, cymes, small flowers, white and spreading sepals, and glabrous stamens; however, it is distinctly different from them in habitat, texture and indumentum of leaflet blades, and sepal apex shape (Figs 5–7).

### ﻿PCA

**Figure 4.**
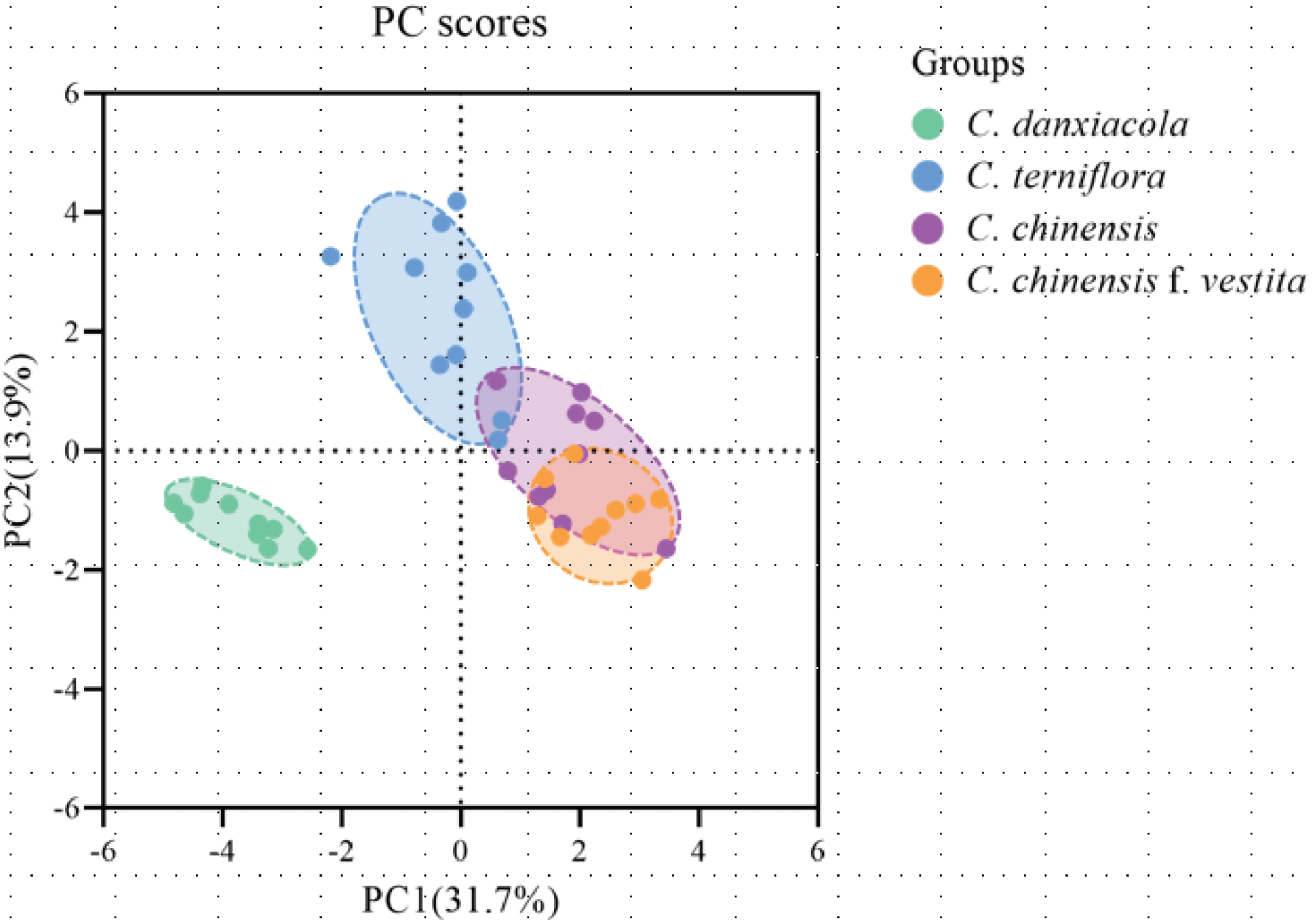
Principal component axes 1 and 2 representing morphological data of *C. danxiacola* sp. nov. and three other similar species.

Multivariate analysis provided a quantitative assessment of morphological differentiation among the four species. The analysis revealed distinct variation patterns along the first two axes (Fig. 4). Two clearly separated clusters emerged: one comprising all specimens of *C. danxiacola* sp. nov., and another containing its allied species. *C. danxiacola* demonstrated a discontinuous distribution in the principal component (PC) 1–PC2 morphospace compared with the other three taxa, forming statistically distinct clusters (Fig. 4).

PCA revealed that the first seven principal components derived from the 20 morphological traits collectively explained 85.16% of the total variation. PC1 accounted for 31.72% of the variance, while PC2 explained 13.93%.

The primary traits associated with PC1 included specimen color after drying, sepal apex characteristics, habitat preferences, fresh leaf taste, and sepal width. PC2 was predominantly influenced by leaf abaxial surface indumentum, fruit length, sepal length, and petiolule length (Table 2).

### ﻿Taxonomic treatment

#### ﻿ Clematis danxiacola

Taxon classificationPlantaeRanunculalesRanunculaceae

J.P.Li, P.Li & Z.H.Chen sp. nov.

BE9642EF-85BA-5797-9DF4-9A3E9078CE64

urn:lsid:ipni.org:names:77371642-1

Figs 5, 6, 7

##### Type.

**China** • Zhejiang Province, Jinhua City, Yongkang County, Zhiying Town, Xianling Village, Chiyantang Reservoir, purple glutenite in the Danxia landform area, in bushes at the forest margin at foothills, alt. 172 m a.s.l., 28°54'29.28"N, 120°8'7.86"E, August 18, 2023, *He-Ping Chen*, *Jun-Ping Li et Zheng-Hai Chen YK23081807* (holotype: ZM barcode ZMNH0067436! isotypes: HZU!, KUN!, PE!).

##### Diagnosis.

This new species resembles *C. terniflora* but differs in several characteristics including leaflet blades abaxially densely persistently appressed-puberulous (*vs.* sparsely puberulous and glabrescent), sepals cuneate or oblanceolate, apex subtruncate and premorse (*vs.* obovate–oblong to oblong, apex ± acute to obtuse and entire), pistils (5–) 8–12 (*vs.* 4–7), achene ovate, compressed but slightly swollen in the middle, margin not or slightly thickened (*vs.* broadly elliptic to obovate, strongly compressed, margin distinctly thickened).

##### Morphological description.

**Figure 5.**
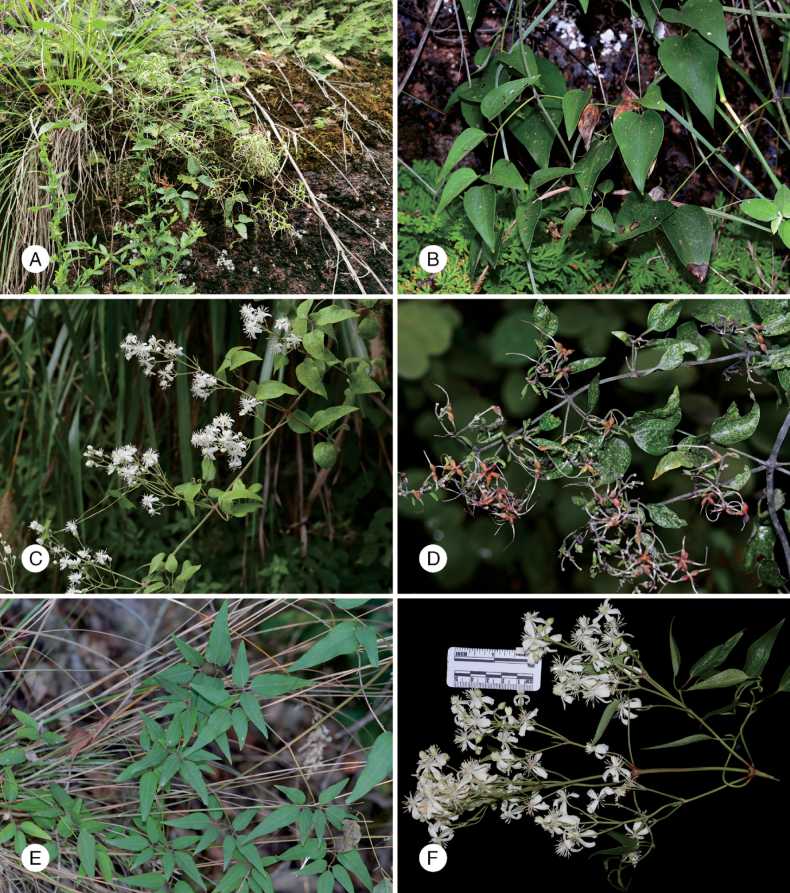
Habitat and habitus of *C. danxiacola* sp. nov. A. Habitat; B. Leaves of spring new shoot; C. Inflorescence of spring new shoot; D. Infructescence of spring new shoot; E. Branchlet and leaves of summer–fall new shoot; F. Inflorescence of summer–fall new shoot. Images captured by Dong-Hao Wu (A), Zheng-Hai Chen (B–E), Jun-Ping Li (F).

**Figure 6.**
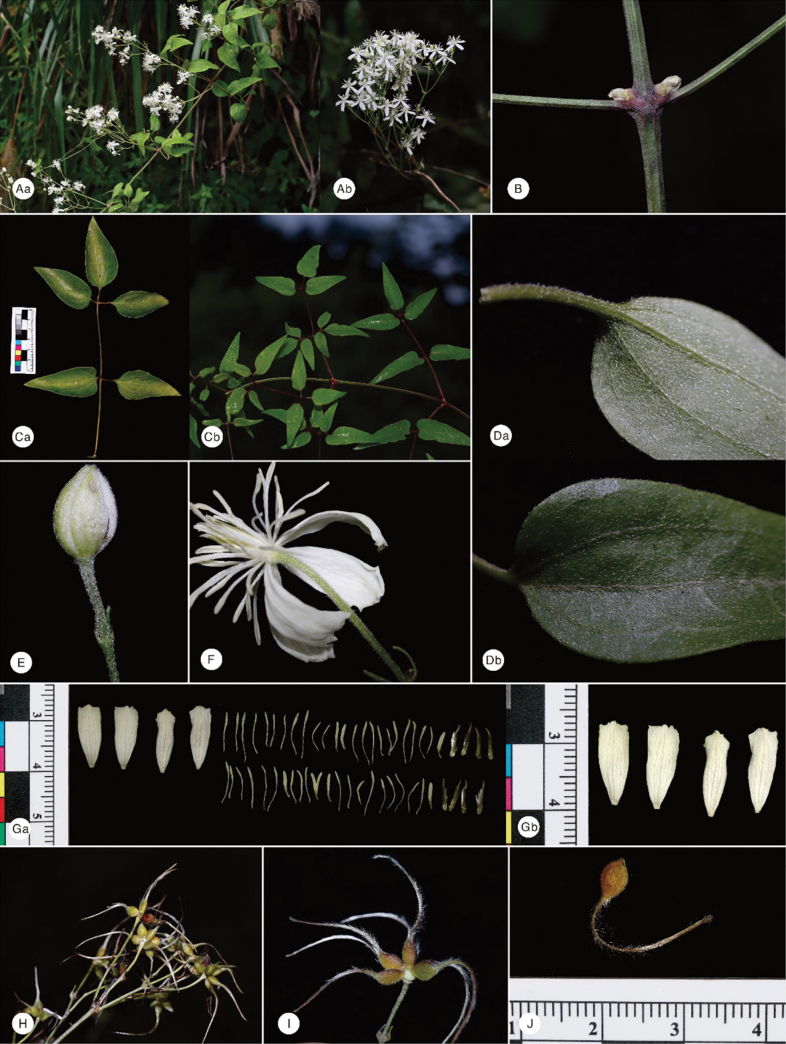
Morphological characteristics of *C. danxiacola* sp. nov. A. Flowering branch (b, summer–fall new shoot); B. Branchlet and axillary bud; C. Leaf (a, spring new shoot; b, summer–fall new shoot); D. Leaflet blade; E. Bud; F. Flower; G. Dissected flower (showing sepals); H. Fruiting branch; I. Infructescence; J. Achene and persistent style. Images captured by Zheng-Hai Chen (A, B, Cb, D–F, H, I) in the field; Jun-Ping Li (Ca, G, J) in the laboratory.

**Figure 7.**
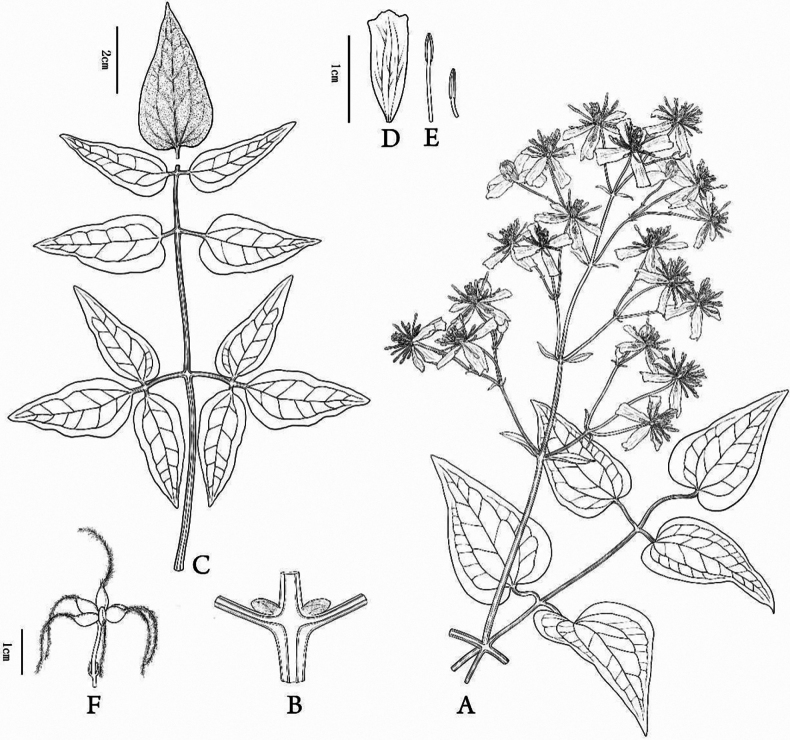
Line drawing of *Clematis danxiacola* sp. nov. A. Flowering branch; B. Stem; C. Leaf; D. Sepal; E. Stamen; F. Infructescence. Drawn by Yi-Chen Wang.

Evergreen woody vine. Stem with shallowly 6–12-grooved; branchlets densely gray appressed-puberulous, glabrescent except at nodes; axillary buds triangular–ovate, densely gray puberulous. Leaves opposite, 1-pinnate, usually 5-foliolate, sometimes 2-pinnate on new shoots in summer–fall; petiole 3–7 cm long, base with nodes usually dark purple or gray, puberulous; leaflet blades thick-papery or subleathery, ovate, 3.5–8.0 (–9.5) × 2.0–5.0 (–6.2) cm, apex acuminate, base broadly cuneate, subrounded to shallow cordate, margin entire, slightly revolute when drying, adaxially appressed-puberulous when young, glabrescent except for basal veins, lustrous, basal veins slightly prominent, abaxially densely persistently appressed-puberulous, basal veins prominent when dry; leaflets of summer–fall shoots thick leathery, ovate–lanceolate or lanceolate, 2.5–10.0 × 1–3 cm, apex long acuminate, base cuneate; petiolules 0.7–2.0 cm long, base usually dark purple, sometimes tendrilous. Paniculate cymes axillary or terminal, usually many-flowered, 5–15 (–30) cm long; bracts usually narrowly lanceolate to linear, 0.7–1.5 (–2.3) × 1.5–4.0 mm, petiolate, rarely leaflike, both surfaces appressed-puberulous. Flowers 2–3 cm in diameter, pungent aromatic, bisexual, erect; buds ovoid, apex acute. Pedicel 1–2 cm long, together with peduncle and rachis densely persistently appressed-puberulous. Sepals 4, white, spreading, cuneate or oblanceolate, 10–14 × 4–6 mm, apex subtruncate and premorse, adaxially glabrous, abaxially densely puberulous, margin velutinous. Stamens 26–49, white, glabrous, 6–11 mm long; filaments 3–7 mm long; anthers linear, 2–4 mm long, apex obtuse or minutely apiculate. Pistils (5–) 8–12; ovaries pubescent; style densely villous. Achenes usually 3–7, brown, ovate, compressed but slightly swollen in the middle, 5.4–6.3 × 3.0–3.7 mm, densely ascending pubescent, margin not or slightly thickened; persistent styles 2.5–3.0 cm long, plumose, whitish (Figs 5–7).

##### Phenology.

Flowering occurs from May to November, with fruiting from September to January of the following year.

##### Etymology.

The specific epithet refers to the Danxia landform where the species occurs.

##### Distribution and ecology.

**Figure 8.**
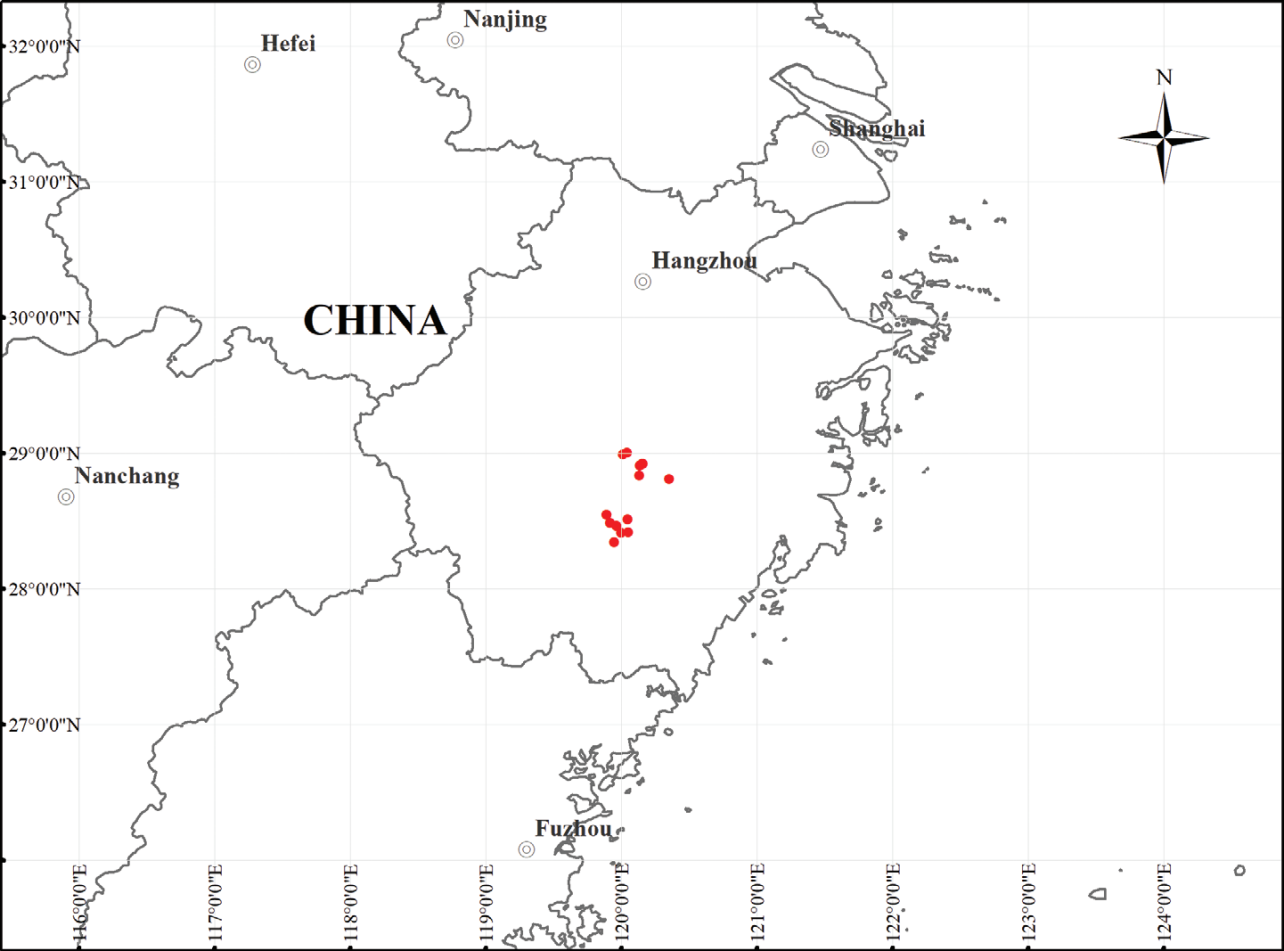
Geographic distribution of *Clematis danxiacola* in Zhejiang Province (red dots).

*C. danxiacola* occurs in Yongkang County of Jinhua City, and Qingtian County, Jinyun County, and Liandu District of Lishui City, Zhejiang Province, China (Fig. 8). The species inhabits sunny slopes or ridges of low hills of the Danxia landform within the elevation range 50–550 m a.s.l, typically climbing on tree canopies or shrubs at forest edges or roadsides. The substrate consists of soil derived from weathered purple sandy conglomerate.

##### Conservation assessment.

The currently known 19 populations of *C. danxiacola* are distributed across two prefecture-level cities, four counties, and 14 towns. The total distribution area is approximately 100 km^2^ with an estimated population exceeding 1000 mature individuals. Despite occurring in low-hill areas characterized by frequent human activities and habitat fragmentation, the species demonstrates resilience due to its drought tolerance, arid soil adaptability, climbing capabilities, high reproductive success, and extensive potential habitat availability. Furthermore, the species currently faces minimal anthropogenic pressure as its economic value remains undetermined, resulting in limited local exploitation. Based on these factors, we propose that *C. danxiacola* be classified as a “Least Concern (LC)” species according to IUCN Red List criteria (IUCN 2024).

##### Additional specimens examined (paratypes).

China • Zhejiang Province, **Jinhua City**, **Yongkang County**, Xicheng Subdistrict, Feilongshan, purple glutenite in the Danxia landform area, the forest margin on slope, alt. 182 m a.s.l., June 29, 2023, *Jun-Ping Li*, *Jian-Ping Zhong et Zheng-Hai Chen YK23062907* (ZM); • same locality, October 9, 2023, *Jun-Ping Li*, *Shang-Jiao Ying et Zheng-Hai Chen YK23100904* (ZM); • same locality, alt. 181 m a.s.l., 28°59'23.88"N, 120°0'41.40"E, August 18, 2023, *Zheng-Hai Chen*, *Jun-Ping Li et He-Ping Chen YK23081801* (ZM, HZU); • Xiangzhu Town, Wangxitian Village, Longqingkeng, purple glutenite in the Danxia landform area, on open land at the forest margin on ridge, alt. 246 m a.s.l., 29°0'8.40"N, 120°2'33.06"E, August 18, 2023, *Shang-Jiao Ying et Xia-Shuo Lei YK23081802* (ZM, HZU); • same locality and date, *Jun-Ping Li*, *He-Ping Chen et Zheng-Hai Chen YK23081808* (ZM); • Zhiying Town, Xianling Village, Chiyantang Reservoir, purple glutenite in the Danxia landform area, on bushes at the forest margin on slope, alt. 199 m a.s.l., July 13, 2023, *Jun-Ping Li YK23071302* (ZM); • same locality, alt. 177 m a.s.l., October 8, 2023, *Jun-Ping Li et Zheng-Hai Chen YK23100801* (ZM); • same locality and date, on ridge bushes, alt. 256 m a.s.l., October 8, 2023, *Jun-Ping Li et Zheng-Hai Chen YK23100802* (ZM); • same locality and date, on ridge bushes, alt. 270 m a.s.l., October 8, 2023, *Jun-Ping Li et Zheng-Hai Chen YK23100803* (ZM); • same locality and date, on bushes near the hilltop, alt. 277 m a.s.l., October 8, 2023, *Jun-Ping Li et Zheng-Hai Chen YK23100804* (ZM); • same locality and date, on ridge bushes, alt. 271 m a.s.l., October 8, 2023, *Jun-Ping Li et Zheng-Hai Chen YK23100805* (ZM); • Qianshanyangxia Village, purple glutenite in the Danxia landform area, on ridge bushes, alt. 236 m a.s.l., 28°55'15.13"N, 120°9'9.49"E, November 8, 2023, *Zheng-Hai Chen*, *Jun-Ping Li et Wen-Yuan Xie YK23110802* (ZM); • same locality and date, alt. 268 m a.s.l., 28°55'10.71"N, 120°9'32.15"E, *Zheng-Hai Chen*, *Jun-Ping Li et Wen-Yuan Xie YK23110808* (ZM); • Shizhu Town, Qianlang Village, purple glutenite in the Danxia landform area, on bushes at the forest margin on slope, alt. 175 m a.s.l., 28°49'59"N, 120°7'14.50"E, August 18, 2023, *Jun-Ping Li*, *He-Ping Chen et Zheng-Hai Chen YK23081805* (ZM); • same locality and date, on bushes at the forest margin on ridge, alt. 230 m a.s.l., *He-Ping Chen*, *Jun-Ping Li et Zheng-Hai Chen YK23081806* (ZM); • same locality, November 7, 2023, *Zheng-Hai Chen*, *Jun-Ping Li et Wen-Yuan Xie YK23110702* (ZM). • **Lishui City**, **Liandu District**, Nanmingshan Subdistrict, Jinshanxia Village, Duimianshan, purple glutenite in the Danxia landform area, in bushes beside the highway, alt. 160 m a.s.l., July 23, 2023, *Dong-Hao Wu LD23072301*, *LD23072302*, *LD23072303* (ZM); • Baiyun Subdistrict, Baiyunshan, Nanbenjishan, purple glutenite in the Danxia landform area, in bushes by a trail, alt. 176 m a.s.l., 28°28'54.4"N, 119°55'4.71"E, July 29, 2023, *Dong-Hao Wu LD23072901* (ZM); • same locality, August 10, 2023, *Wen-Yuan Xie*, *Jun-Feng Wang*, *Zheng-Hai Chen* et al. *LD23081001* (ZM, HZU); • Beibenjishan, purple glutenite in the Danxia landform area, in roadside bushes at the mountain foot, alt. 155 m a.s.l., July 29, 2023, *Dong-Hao Wu LD23072902* (ZM); • Zijin Subdistrict, Hebian Village, Bogushan, purple glutenite in the Danxia landform area, in roadside bushes at the mountain foot, alt. 90 m a.s.l., August 5, 2023, *Jian-Ping Zhong et Dong-Hao Wu LD23080501* (ZM); • Huangnidan Village, purple glutenite in the Danxia landform area, on bushes amid steep slopes near the highway, alt. 98 m a.s.l., 28°27'37.23"N, 119°57'47.73"E, August 5, 2023, *Jian-Ping Zhong et Dong-Hao Wu LD23080502* (ZM); • the same locality, August 10, 2023, *Wen-Yuan Xie*, *Jun-Feng Wang*, *Zheng-Hai Chen* et al. *LD23081003* (ZM, HZU); • the same locality, October 10, 2023, *Jun-Feng Wang et Zheng-Hai Chen LD23101001* (ZM); • Shuilinggen Village, purple glutenite in the Danxia landform area, on bushes near the highway, alt. 333 m a.s.l., August 5, 2023, *Jian-Ping Zhong et Dong-Hao LD23080503* (ZM); • Fuling Subdistrict, Jinshan, purple glutenite in the Danxia landform area, beside a hiking trail on the hillside, alt. 154 m a.s.l., October 10, 2023, *Jun-Feng Wang et Zheng-Hai Chen LD23101002* (ZM); • the same locality and date, on tree crown by the hillside road, alt. 160 m a.s.l., *Jun-Feng Wang et Zheng-Hai Chen LD23101003* (ZM); • the same locality and date, on tree crown by the hillside road, alt. 165 m a.s.l., *Jun-Feng Wang et Zheng-Hai Chen LD23101005* (ZM); • the same locality and date, on bushes by the hillside road, alt. 163 m a.s.l., *Jun-Feng Wang et Zheng-Hai Chen LD23101006* (ZM); • the same locality and date, on bushes by the hillside path, alt. 162 m a.s.l., *Jun-Feng Wang et Zheng-Hai Chen LD23101007* (ZM); • Huangcun Town, Huangcun Village, purple glutenite in the Danxia landform area, in bushes by the highroad near a small temple, alt. 129 m a.s.l., 28°30'40.02"N, 120°2'48.74"E, July 30, 2023, *Dong-Hao Wu LD23073001* (ZM); • Yanquan Subdistrict, introduced from Xiayinkeng Village and cultivated by *Yong-Jun Chen*, alt. 50 m a.s.l., July 30, 2023, *Dong-Hao Wu LD23073002* (ZM); • the same locality, Xiayinkeng Village, Changkeng, purple glutenite in the Danxia landform area, in bushes by the roadside, alt. 235 m a.s.l., August 4, 2023, *Dong-Hao Wu et Yong-Jun Chen LD23080401* (ZM); • Taiping Town, on steep shrubs, 119°53'19.8"E, 28°32'34.0"N, alt. 453 m a.s.l., July 27, 2024, *Lian-Hai Wu* et *Zheng-Hai Chen LD24072701* (ZM); • the same locality and date, 119°53'19.87"E, 28°32'34.09"N, alt. 410 m a.s.l., *Jun-Feng Wang* et *Dong-Hao Wu 24072702* (ZM). • **Jinyun County**, Huzheng Town, on steep shrubs, 120°21'12.12"E, 28°48'18.63"N, alt. 550 m a.s.l., July 7, 2024, *Shang-Jiao Ying*, *Jun-Ping Li*, *Liang-Dong>* et *Zheng-Hai Chen JY24070701* (ZM). • **Qingtian County**, Lakou Town, near Kengkou Village, on shrubs and on rock walls next to the highway, 28°20'31.55"N, 119°56'53.19"E, alt. 53 m a.s.l., August 17, 2024, *Jun-Feng Wang*, *Dong-Hao Wu* et *Jian-Ping Zhong QT24081701* (KUN, PE, ZM); • the same locality and date, near Yaojun Village, 28°24'31.26"N, 119°59'57.36"E, alt. 102 m a.s.l., *Dong-Hao Wu* et *Jian-Ping Zhong QT24081702* (ZM); • Shuqiao Town, near Shakeng Village, on a tree crown and on a rock wall by the roadside, 28°24'54.20"N, 120°2'51.26"E, alt. 277 m a.s.l., *Jun-Feng Wang* et *Jian-Ping Zhong QT24081703* (ZM).

## ﻿Discussion

The new species *C. danxiacola* exhibits close phylogenetic and morphological relationships to *C. terniflora* and *C. chinensis*. In the reconstructed phylogenetic tree, *C. danxiacola* is positioned within sect. Clematis (Wang and Li 2005), which is characterized by opposite phyllotaxy, spreading calyx, and glabrous filaments. This study corroborates previous phylogenetic analyses of *Clematis* that grouped *C. terniflora*, *C. chinensis*, *C. armandii* Franch. (1885: 184), *C. crassifolia* Benth. (1861: 7), *C. hexapetala* Pall. (1776: 735), and *C. brachyura* Maxim. (1877: 221) as a monophyletic group (Xie et al. 2011; Xiao et al. 2022). The phylogenetic placement of *C. danxiacola* within the clade demonstrates slight variations between plastome and ITS sequences. In the plastome phylogenetic tree, *C. danxiacola* specimens form a clade sister to that containing *C. terniflora* and *C. chinensis*, while in the ITS phylogenetic tree, *C. danxiacola* formed a clade with *C. armandii*, and this combined clade was sister to a clade comprising *C. terniflora*, *C. mandshurica* Rupr. (1857: 258), *C. chinensis*, and *C. hexapetala*. This cyto-nuclear discordance aligns with previous research findings, suggesting widespread interspecific hybridization events in *Clematis* (Xiao et al. 2022). Morphologically, *C. danxiacola* shares the strongest similarities with *C. terniflora*; it bears some resemblance to *C. chinensis* and C. chinensis var. vestita (Rehder & E.H.Wilson) W.T. Wang (1998: 158) [≡ C. chinensis f. vestita Rehder & E.H.Wilson (1913: 330)], but maintains its natural color when dried, and exhibits distinct characteristics in its bracts and sepals (Table 3).

**Table 3.** Morphological comparison with *Clematis danxiacola* sp. nov. and its allied taxa.

Characters	*C. danxiacola* sp. nov. ^ζ^	*C. terniflora* ^α, β^	C. chinensis var. chinensis ^α, γ^	C. chinensis var. vestita ^α, δ, ε^
Plant on drying	not turning black	not turning black	turning bright black	turning black
Branchlet	densely persistently appressed-puberulous; nodes with base of petiole and petiolules usually dark purple	puberulous; nodes with petiole and petiolules usually green	glabrous or sparsely puberulous; nodes with petiole and petiolules usually green	± densely puberulous
Leaf	1-pinnate, 5-foliolate; 1- or 2-pinnate on new shoots in summer-autumn	1-pinnate, 5 (–7)-foliolate	1-pinnate, 5-foliolate	1-pinnate, 5-foliolate
leaflet blade	thick-papery or subleathery, 3.5–8.0 (–9.5) × 2.0–5.0 (–6.2) cm, abaxially densely persistently appressed-puberulous,	papery to subleathery, 2.5–8 × 1–4.2 cm, both surfaces sparsely puberulous, glabrescent	papery, 1.5–9.5 × 0.7–6.4 cm, both surfaces subglabrous or very sparsely puberulous only on basal veins	3.5 (–5) × 2 (–2.5) cm, abaxially ± densely puberulous
Bract	narrowly lanceolate to linear, 0.7–1.5 (–2.3) cm long, petiolate, rarely leaflike	linear, elliptic or oblong, 0.8–3.5 (–5.0) cm long	petiolate and elliptic to oblong or sessile, small, and linear	petiolate and elliptic to oblong or sessile, small, and linear
Pedicel	1–2 cm long, densely persistently appressed-puberulous	0.5–3.0 cm long, puberulous	1.4–3.0 cm long, sparsely puberulous	1.4–3.0 cm long, sparsely puberulous
Sepal	cuneate or oblanceolate, 10–14 × 4–6 mm, apex subtruncate and premorse	obovate-oblong to oblong, 5–15 × 2–6 mm, apex acute to obtuse and entire	obovate-oblong, oblanceolate, or lanceolate, 6–13 × 1.8–3 (–4) mm, apex acute and entire	obovate-oblong, oblanceolate, 6–9 × 2–3 mm, apex acute and entire
Pistil	(5–) 8–12	4–7	—	—
Achene	ovate, 5.4–6.3 × 3.0–3.7 mm, compressed and slightly swollen on middle both sides, margin not or slightly thickened	broadly elliptic to obovate, 6–9 × 3–6 mm, strongly compressed, margin distinctly thickened	elliptic, 5–7 × 3.5–4 mm, appressed puberulous	—
Habitat	Danxia landform, low hills, purple sandstone and conglomerate, arid habitats, alt. 50–550 m a.s.l.	various landforms, humid habitats, alt. below 600 m a.s.l.	various landforms, humid habitats, alt. 100–1500 m a.s.l.	thickets, alt. 300–1100 m a.s.l.

Based on Wang and Bartholomew (2001) α, DC. (1818) β, Osbeck (1757) γ, Rehder and Wilson (1913) δ, Wang (1998) ε, and this paper (measurements at ZM) ζ; ——data deficient.

## ﻿Conclusion

This study presents a new species of *Clematis* based on morphological and molecular evidence. The documented plastome provides valuable data for future research on the systematics, evolution, and conservation of the genus.

## Supplementary Material

XML Treatment for Clematis danxiacola

## ﻿Additional information

### Use of AI

No use of AI was reported.

### Author ORCIDs

Jun-Ping Li https://orcid.org/0009-0000-7655-3849

Qing Ma https://orcid.org/0000-0002-2322-8847

Dan-Dan Ma https://orcid.org/0000-0002-7273-9141

Jun-Feng Wang https://orcid.org/0000-0002-8110-9045

Wen-Yuan Xie https://orcid.org/0000-0003-1529-0787

Dong-Hao Wu https://orcid.org/0009-0003-7113-6554

Shang-Jiao Ying https://orcid.org/0009-0005-7201-5775

Zheng-Hai Chen https://orcid.org/0000-0002-8852-7127

Pan Li https://orcid.org/0000-0002-9407-7740

### Data availability

All of the data that support the findings of this study are available in the main text or Supplementary Information.

## Supplementary materials

Supplementary material 1 Supplementary tables S1, S2 Data type docx https://binary.pensoft.net/file/1459612This dataset is made available under the Open Database License (http://opendatacommons.org/licenses/odbl/1.0/). The Open Database License (ODbL) is a license agreement intended to allow users to freely share, modify, and use this Dataset while maintaining this same freedom for others, provided that the original source and author(s) are credited.Jun-Ping Li, Qing Ma, Dan-Dan Ma, Jun-Feng Wang, Wen-Yuan Xie, Dong-Hao Wu, Shang-Jiao Ying, Zheng-Hai Chen, Pan Li

## References

[B1] Bankevich A Nurk S Antipov D Gurevich AA Dvorkin M Kulikov AS Lesin VM Nikolenko SI Pham S Prjibelski AD (2012) SPAdes: A new genome assembly algorithm and its applications to single-cell sequencing. Journal of Computational Biology 19: 455–477. 10.1089/cmb.2012.0021 22506599 PMC3342519

[B2] Bentham G (1861) Flora Hongkongensis: A Description of the Flowering Plants and Ferns of the Island of Hongkong. Lovell Reeve, London, 7 pp. 10.5962/bhl.title.21052

[B3] Brandenburg WA (2000) Meclatis in Clematis: Yellow flowering *Clematis* species. Systematic studies in Clematis I. (Ranunculaceae), inclusive of cultonomic aspects. Wageningen Universiteit, Wageningen.

[B4] Chen LY Song MS Zha HG Li ZM (2014) A modified protocol for plant genome DNA extraction. Plant Diversity and Resources 36: 375–380.

[B5] de Candolle AP (1818) Regni vegetabilis systema naturale I. Treuttel & Wurtz, Paris, 137 pp. 10.5962/bhl.title.59874

[B6] Franchet A (1885) PlantaeDavidianae ex Sinarum Imperio: Plantes du Thibet Oriental, Province de Moupine. Nouvelles Archives du Muséum d’Histoire Naturelle, sér. 2, 8: 184.

[B7] Greiner S Lehwark P Bock R (2019) OrganellarGenomeDRAW (OGDRAW) version 1.3.1: Expanded toolkit for the graphical visualization of organellar genomes. Nucleic Acids Research 47: W59–W64. 10.1093/nar/gkz238 PMC6602502 30949694

[B8] Greuter W (1965) Beiträge zur Flora der Südägäis 1–7. Candollea 20: 212–213. 10.5169/seals-880361

[B9] Grey-Wilson C (2000) *Clematis* the genus. Timber Press, Portland, 223 pp.

[B10] He J Lyu RD Luo YK Lin LL Yao M Xiao JM Xie L Wen J Pei LY Yan SX Cheng J Li JY Li LQ (2021) An updated phylogenetic and biogeographic analysis based on genome skimming data reveals convergent evolution of shrubby habit in *Clematis* in the Pliocene and Pleistocene. Molecular Phylogenetics and Evolution 164: 1–14. 10.1016/j.ympev.2021.107259 34303792

[B11] IUCN (2024) Guidelines for Using the IUCN Red List Categories and Criteria. Version 16. Prepared by the Standards and Petitions Committee, Gland, Switzerland. http://www.iucnredlist.org/documents/RedListGuidelines.pdf [accessed 15 January 2025]

[B12] Jiang N Zhou Z Yang JB Zhang SD Guan KY Tan YH Yu WB (2017) Phylogenetic reassessment of tribe anemoneae Ranunculaceae): Non-monophyly of *Anemone* s. l. revealed by plastid datasets. PLOS ONE 12: 1–17. 10.1371/journal.pone.0174792 PMC5376084 28362811

[B13] Jin JJ Yu WB Yang JB Song Y Yi TH Li DZ (2020) GetOrganelle: A fast and versatile toolkit for accurate de novo assembly of organelle genomes. Genome Biology 21: 241. 10.1186/s13059-020-02154-5 PMC7488116 32912315

[B14] Johnson M (1997) Släktet Klematis. Magnus Johnson Plantskola AB, Södertälje, 881 pp.

[B15] Katoh K Standley DM (2013) MAFFT multiple sequence alignment software version 7: Improvements in performance and usability. Molecular Biology and Evolution 30(4): 772–780. 10.1093/molbev/mst010 23329690 PMC3603318

[B16] Linnaeus C (1753) Species Plantarum. Laurentius Salvius, Stockholm, 1200 pp.

[B17] Maximowicz CJ (1877) Diagnoses plantarum novarum Japoniae et Mandshuriae. Bulletin de l’Académie Impériale des Sciences de Saint-Pétersbourg 22: 221. 10.5962/bhl.title.46308

[B18] Miikeda O Kita K Handa T Yukawa T (2006) Phylogenetic relationships of *Clematis* (Ranunculaceae) based on chloroplast and nuclear DNA sequences. Botanical Journal of the Linnean Society 152: 153–168. 10.1111/j.1095-8339.2006.00551.x

[B19] Osbeck P (1757) Dagbok öfwer en Ostindisk resa åren 1750, 1751, 1752: Med anmårkningar uti naturkunnigheten, fråmmande folkslags språk, seder, hushållning, m. m. Stockholm, Lor. Ludv. Grefing, 205 pp. 10.5962/bhl.title.112527

[B20] Pallas PS (1776) Reise durch verschiedene Provinzen des russischen Reichs 3. St. Petersburg, Kaiserliche Akademie der Wissenschaften, 735 pp.

[B21] Rehder A Wilson EH (1913) *Clematis*. In: Sargent CS (Ed.) PlantaeWilsonianae 1. The University Press, Cambridge, 330 pp.

[B22] Ruprecht FJ (1857) Die ersten botanischen Nachrichten über das Amurland. Zweite Abthkilung: Bäume und Sträucher, Bulletin de la Classe Physico-Mathematique de l’Academie Imperiale des Sciences de Saint-Pétersbourg. St. Petersburg ser. 2, 15: 258.

[B23] Stamatakis A (2014) RAxML Version 8: A tool for phylogenetic analysis and post-analysis of large phylogenies. Bioinformatics (Oxford, England) 30(9): 1312–1313. 10.1093/bioinformatics/btu033 24451623 PMC3998144

[B24] Tamura M (1995) *Clematis*. In: Hiepko P (Ed.) Die Natürlichen Pflanzenfamilien.2^nd^ ed. 17a. Duncker & Humblot, Berlin, 368–387.

[B25] Thiers B (2024 onwards) Index Herbariorum. http://sweetgum.nybg.org/science/ih/ [accessed on 13 December 2024]

[B26] Wang WT (1980) Flora Reipublicae Popularis Sinicae 28. Science Press, Beijing, 75–235.

[B27] Wang WT (1998) Notulae de Ranunculaceis Sinensibus (XXII). Zhiwu Fenlei Xuebao 36: 150–172. https://www.jse.ac.cn/EN/Y1998/V36/I2/150

[B28] Wang WT (2003) A revision of Clematis sect. Clematis (Ranunculaceae). Zhiwu Fenlei Xuebao 41(1): 1–62. 10.1360/aps050049

[B29] Wang WT Bartholomew B (2001) *Clematis* L. In: Wu CY Raven P (Eds) Flora of China 6.Science Press, Beijing & Missouri Botanical Garden Press, St. Louis, 333–386.

[B30] Wang WT Li LQ (2005) A new system of classification of the genus *Clematis* (Ranunculaceae). Zhiwu Fenlei Xuebao 43(5): 431–488. 10.1360/aps040091

[B31] Xiao JM Lyu RD He J Li MY Ji JX Cheng J Xie L (2022) Genome-partitioning strategy, plastid and nuclear phylogenomic discordance, and its evolutionary implications of *Clematis* (Ranunculaceae). Frontiers in Plant Science 13: 1–14. 10.3389/fpls.2022.1059379 PMC9703796 36452086

[B32] Xie L Wen J Li LQ (2011) Phylogenetic analyses of *Clematis* (Ranunculaceae) based on sequences of nuclear ribosomal ITS and three plastid regions. Systematic Botany 36(4): 907–921. 10.1600/036364411X604921

[B33] Ziman SN Keener CS (1989) A geographical analysis of the family Ranunculaceae. Annals of the Missouri Botanical Garden 76(4): 1012–1049. 10.2307/2399690

