# Supplementary material for: ﻿Clematis danxiacola (Ranunculaceae), a new species from the Danxia landform area in Zhejiang Province, China
Source: PhytoKeys. 2025 Nov 7;266:97–115. doi: 10.3897/phytokeys.266.154626 (PMC12619068; doi:10.3897/phytokeys.266.154626)
Supplement: Supplementary material 1 — Supplementary tables S1, S2 [file phytokeys-266-097_article-154626__-s001.docx]

**Table S1. Species, accession numbers, and phylogenetic classification of species used in this study.**

| Species | GenBank No.for cpDNA | GenBank No.for ITS | Clade |
| --- | --- | --- | --- |
| *Clematis reticulata* | MT876512 | GU732625 | VI |
| *Clematis glaucophylla* | NC_065274 | MT894320 | - |
| *Clematis crispa* | NC_065269 | KR909795 | VI |
| *Clematis huchouensis* | NC_065277 | KR909784 | - |
| *Clematis fusca* | NC_060524 | AB120179 | VI |
| *Clematis florida* | NC_058885 | AB120186 | - |
| *Clematis cadmia* | MT796624 | KR909771 | - |
| *Clematis integrifolia* | NC_081053 | GU732598 | VI |
| *Clematis xiangguiensis* | MT876521 | MT894354 | - |
| *Clematis pubescens* | MT876509 | GU732620 | III |
| *Clematis linearifolia* | PQ874732 | PQ874732 | III |
| *Clematis petriei* | MT876507 | MT894335 | - |
| *Clematis smilacifolia* | PV423350 | - | - |
| *Clematis elchleri* | - | AB120209 | IX |
| *Clematis loureiroana* | KY083057 | KR909782 | IX |
| *Clematis chinensis* | OQ992636, OQ980378, OR027027 | AB775173, KY201178, AB775174 | V |
| *Clematis terniflora* | MZ959066, NC_028000 | MH711083, AB775157, GU732608, KP997255 | V |
| *Clematis brachyura* | MH104710 | - | V |
| *Clematis mandshurica* | OK375873 | KR082764 | V |
| *Clematis hexapetala* | OP369301 | GU732597 | V |
| *Clematis taeguensis* | NC_054335 | - | - |
| *Clematis lancifolia* | NC_065279 | MT894326 | - |
| *Clematis armandii* | NC_069848 | GU732578 | V |
| *Clematis crassifolia* | NC_065268 | AB120194 | - |
| *Clematis quinquefoliolata* | NC_050373 | MT894340 | - |
| *Clematis uncinata* | NC_081060 | AB120189 | - |
| *Clematis rutiodes* | NC_065289 | GU732626 | IV |
| *Clematis aureolata* | NC_069849 | PQ842561 | - |
| *Clematis stans* | ON411442 | AB120188 | VII |
| *Clematis speciosa* | NC_081062 | ON644582 | - |
| *Clematis urticifolia* | NC_081060 | ON644603 | - |
| *Clematis psilandra* | ON411439 | ON644581 | - |
| *Clematis tsugetorum* | NC_081059 | ON644591 | - |
| *Clematis tubulosa* | MT796601 | KR909783 | - |
| *Clematis pinnata* | MT796600 | GU732616 | - |
| *Clematis heracleifolia* | MT796606 | GU732596 | VII |
| *Clematis brevicaudata* | MG675223 | GU732583 | VII |
| *Clematis serratifolia* | NC_060523 | MG235132 | - |
| *Clematis gratopsis* | NC_081052 | FJ424226 | - |
| *Clematis trichotoma* | NC_043828 | - | - |
| *Clematis virginiana* | MT876518 | GU732639 | VII |
| *Clematis campestris* | NC_065264 | MT894310 | - |
| Species | GenBank No.for cpDNA | GenBank No.for ITS | Clade |
| *Clematis haenkeana* | NC_065275 | MT894321 | - |
| *Clematis drummondii* | NC_065271 | GU732591 | VII |
| *Clematis ligusticifolia* | NC_065282 | AB120201 | VII |
| *Clematis vitalba* | NC_081061 | AB120207 | VII |
| *Clematis peterae* | NC_081055 | GU732614 | VII |
| *Clematis subumbellata* | ON411444 | ON644587 | VII |
| *Clematis delavayi* | NC_065270 | AB120202 | VII |
| *Clematis gouriana* | NC_081050 | KX277658 | - |
| *Clematis apiifolia* | NC_081049 | GU732577 | VII |
| *Clematis lasiandra* | MT876510 | AB120200 | VIII |
| *Clematis urophylla* | MT876517 | MT894350 | - |
| *Clematis henryi* | NC_070190 | KR909797 | - |
| *Clematis leschenaultiana* | NC_065281 | KR909785 | - |
| *Clematis grandidentata* | NC_081051 | - | - |
| *Clematis parviloba* | NC_081054 | - | - |
| *Clematis williamsii* | MT876520 | AB120181 | VII |
| *Clematis guniuensis* | NC_081050 | - | - |
| *Clematis alternata* | MG675221 | MT894306 | VIII |
| *Clematis connata* | NC_065267 | MT894313 | - |
| *Clematis rehderiana* | ON520704 | GU732624 | VIII |
| *Clematis brachiata* | MT796623 | GU732579 | VIII |
| *Clematis potaninii* | MW542990 | AB120198 | - |
| *Clematis ranunculoides* | NC_081994 | GU732621 | VIII |
| *Clematis montana* | MT292622 | GU732610 | X |
| *Clematis chrysocoma* | NC_065266 | GU732585 | X |
| *Clematis tomentella* | ON854662 | MT894348 | - |
| *Clematis nannophylla* | MT876504 | - | - |
| *Clematis fruticosa* | NC_065273 | MN722021 | - |
| *Clematis songorica* | MT876515 | KC415712 | - |
| *Clematis canescens* | NC_065265 | KU853294 | - |
| *Clematis viridis* | MT876519 | MT894352 | - |
| *Clematis akebioides* | OR801210 | GU732574 | I |
| *Clematis tibetana* | ON520705 | GU732636 | I |
| *Clematis glauca* | NC_056311 | PV263266 | - |
| *Clematis acerifolia* | NC_039844 | GU732572 | - |
| *Clematis repens* | MG675222 | MT894341 | - |
| *Clematis otophora* | NC_069850 | PQ888929 | - |
| *Clematis macropetala* | MT876503 | JN809684 | - |
| *Clematis alpina* | MT876505 | GU732575 | II |
| *Clematis calcicola* | NC_066977 | - | - |
| *Clematis nobilis* | - | AB120206 | - |
| *Anemoclema glaucifolium* | NC_037194 | KR909768 | - |

*Species were assigned to clades based on the phylogenetic framework of Xie et al. (2011). A dash ("-") indicates that the species was not encompassed by or assigned to one of the ten major clades in the previous study. For cpDNA and ITS GenBank numbers, a dash ("-") denotes that the corresponding sequence was not available or used for phylogenetic reconstruction.

**Table S2. Genes encoded in plastid genome of *Clematis danxiacola* sp. nov*.***

| Category | Group of genes | Genes names |
| --- | --- | --- |
| Self-replication | Transfer RNA gene | *trnA-UGC* (×2)*, *trnI-GAU* (×2)*, *trnI-CAU* (×2), *trnL-CAA* (×2), *trnN-GUU* (×2), *trnR-ACG* (×2), *trnV-GAC* (×2), *trnC-GCA*, *trnK-UUU**, *trnH-GUG*, *trnS-GCU*, *trnL-UAA**, *trnQ-UUG*, *trnF-GAA*, *trnG-UCC**, *trnR-UCU*, *trnD-GUC*, *trnT-GGU*, *trnY-GUA*, *trnE-UUC*, *trnG-GCC*, *trnS-UGA*, *trnfM-CAU*, *trnS-GGA*, *trnV-UAC**, *trnM-CAU*, *trnW-CCA*, *trnP-UGG*, *trnL-UAG* |
|  | Ribosomal RNA genes | *rrn16* (×2), *rrn23* (×2), *rrn4.5* (×2), *rrn5* (×2) |
|  | Small subunit of ribosome | *rps16**, *rps2*, *rps14*, *rps4*, *rps18*, *rps12* (×2)**, *rps11*, *rps8* (×2), *rps3* (×2), *rps19* (×2), *rps7* (×2), *rps15* |
|  | Large subunit of ribosome | *rpl33*, *rpl20*, *rpl36*, *rpl14* (×2), *rpl16* (×2)*, *rpl22* (×2), *rpl2* (×2)*, *rpl23* (×2), *rpl32* |
| Photosynthesis | DNA-dependent RNA polymerase | *poC2*, *rpoC1**, *rpoB*, *rpoA* |
|  | Subunits of photosystem I | *psaB*, *psaA*, *psaI*, *psaJ*, *psaC*, *ycf3*** |
|  | Subunits of photosystem II | *psbA*, *psbK*, *psbI*, *psbM*, *psbD*, *psbC*, *psbZ*, *psbJ*, *psbL*, *psbF*, *psbE*, *psbB*, *psbT*, *psbN*, *psbH* |
|  | Large subunit of rubisco | *rbcL* |
|  | NADH dehydrogenase | *ndhJ*, *ndhK*, *ndhC*, *ndhB* (×2)*, *ndhF*, *ndhD*, *ndhE*, *ndhG*, *ndhI*, *ndhA**, *ndhH* |
|  | Cytochrome b/f complex | *petN*, *petA*, *petL*, *petG*, *petB**, *petD** |
|  | ATP synthase | *atpA*, *atpF**, *atpH*, *atpI*, *atpE*, *atpB* |
| Other genes | Maturase | *matK* |
|  | Subunit of acetyl-CoA carboxylase | *accD* |
|  | Envelope membrane protein | *cemA* |
|  | Protease | *clpP*** |
|  | Translational initiation factor | *infA* (×2) |
|  | C-type cytochrome synthesis | *ccsA* |
|  | Conserved open reading frames | *ycf4*, *ycf2* (×2), *ycf1* |

^#^Genes with one or two introns are indicated by one (*) or two asterisks (**), respectively. Genes in the IR regions are followed by the (×2) symbol.
